# Study protocol for a midwife-led psychoeducational intervention to reduce pregnancy-related anxiety in low-risk pregnant women

**DOI:** 10.3389/fpubh.2026.1832201

**Published:** 2026-06-03

**Authors:** Aleksandra Krawczyk, Agnieszka Czerwińska-Osipiak, Lucyna Wójcicka, Anna Michalik, Agnieszka Gruszecka

**Affiliations:** 1 Division of Obstetric and Gynaecological Nursing, Faculty of Health Sciences With the Institute of Maritime and Tropical Medicine, Medical University of Gdańsk, Gdańsk, Poland; 2 Department of Radiology Informatics and Statistics, Medical University of Gdańsk, Gdańsk, Poland

**Keywords:** fear of childbirth, midwife-led care, perinatal mental health, pregnancy-related anxiety, psychoeducational intervention

## Abstract

**Introduction:**

Pregnancy-related anxiety is a distinct form specifically focused on perinatal concerns, including the course of pregnancy, childbirth and parenthood. Despite its clinical significance, pregnancy-related anxiety remains underrecognised in routine antenatal care. Poland has one of the highest caesarean section rates in Europe, with maternal fear of childbirth and anxiety-driven preferences for operative birth identified as major contributing factors. The aim of this study is to evaluate whether an individual, midwife-led psychoeducational intervention delivered in the second and third trimester reduces pregnancy-related anxiety.

**Materials and methods:**

This is a two-arm, parallel-group randomised controlled trial with a 1:1 allocation ratio. A total of 74 low-risk pregnant women with elevated pregnancy-related anxiety (PRAQ-R2 score ≥30 points) will be recruited from antenatal clinics, childbirth education schools and online platforms. The intervention group will receive four to six individual psychoeducational sessions delivered by a trained midwife between 24 and 34 weeks of gestation, focusing on childbirth education, pain management, communication skills, birth planning and anxiety management techniques. The control group will receive standard antenatal care and an informational booklet. The primary outcome is the reduction in pregnancy-related anxiety levels measured via the Pregnancy-Related Anxiety Questionnaire – Revised 2 between 34 and 37 weeks. Secondary outcomes include actual mode of birth and childbirth satisfaction, as well as maternal and neonatal health outcomes. Data will be collected at three time points and analysed using intention-to-treat principles.

**Discussion:**

This trial will provide initial evidence on the effectiveness of a scalable, midwife-delivered psychoeducational intervention tailored to the Polish perinatal context. If proven effective, the programme could improve maternal psychological wellbeing and strengthen the role of midwives in antenatal mental healthcare. The findings may inform future policy changes and perinatal care standards in Poland and other countries with similarly high caesarean section rates and fragmented maternity care systems.

**Clinical trial registration:**

ClinicalTrials.gov, identifier NCT07323459.

## 1 Introduction

### 1.1 Background and rationale

Pregnancy-related anxiety (PrA) represents a pregnancy-specific form of anxiety, characterised by concerns confined to perinatal matters: the course of pregnancy, approaching labour and delivery, as well as forthcoming parenthood. Evidence suggests that these worries are qualitatively distinct from generalised anxiety disorder (GAD). Whereas GAD manifests as diffuse apprehension across multiple life domains, PrA exclusively addresses pregnancy-related factors—including maternal and foetal wellbeing, pregnancy outcomes and somatic changes experienced during gestation. Despite the absence of PrA as a formal diagnostic category in the Diagnostic and Statistical Manual of Mental Disorders (DSM-5) or the International Classification of Diseases (ICD-11), accumulating empirical evidence supports its conceptualisation as a distinct clinical construct. In clinical practice, its symptoms are commonly classified under broader categories such as other specified anxiety disorder or adjustment disorder, reflecting ongoing attempts to accommodate pregnancy-specific anxiety within existing diagnostic frameworks (1). Notably, mild pregnancy-related anxiety is considered physiological and potentially adaptive, promoting childbirth and parenting preparation. In contrast, severe and persistent anxiety that causes distress may impair expectant mothers’ daily functioning and can adversely impact maternal and foetal and neonatal health (1–6).

#### 1.1.1 Symptoms and areas of pregnancy-related anxiety

Symptoms of pregnancy-related anxiety resemble those observed in other anxiety disorders; however, their content is specifically focused on pregnancy-related concerns. These symptoms may include excessive worry, tension, and cognitive preoccupation with such potential threats. Elevated PrA has been associated with adverse maternal and neonatal outcomes, including preterm birth and increased obstetric interventions (5, 7–10).

Pregnant women’s anxieties are centred on several key areas. Most frequently, they concern the health and life of the child, encompassing fears of miscarriage, pregnancy loss and the possibility of giving birth to a child with an illness or genetic defect, as well as concerns related to the proper development of the foetus and newborn. Another significant source of anxiety is the birth process itself and maternal health, manifesting through intense fear of labour pain, complications during delivery (such as the need for caesarean section or haemorrhage), and concerns about one’s own life and health during this event (2, 11, 12).

Although fear of childbirth (FoC) and pregnancy-related anxiety (PrA) share common determinants and consequences, they represent conceptually distinct constructs (2, 4). Fear of childbirth refers specifically to anxiety associated with labour and delivery, including concerns related to labour pain, obstetric complications, and the birthing process itself (12). In contrast, pregnancy-related anxiety encompasses a broader spectrum of pregnancy-specific concerns (1, 2), including fears related to foetal health, maternal wellbeing, pregnancy outcomes, childbirth, and future parenting responsibilities. While FoC may be considered one component of pregnancy-related anxiety, distinguishing between these constructs is essential for selecting appropriate screening tools and designing targeted interventions (2, 12).

Tokophobia is typically conceptualised as a distinct clinical entity characterised by an intense and persistent fear of childbirth (12, 13). Although it may co-occur with PrA and shares overlapping determinants, tokophobia represents a specific form of childbirth-related fear rather than a severity extreme of pregnancy-related anxiety (2, 6, 12). The psychoeducational approach proposed in this study is therefore relevant for addressing PrA while also supporting women experiencing elevated fear of childbirth (14–16).

#### 1.1.2 Risk factors and consequences of pregnancy-related anxiety

The experience of pregnancy-related anxiety varies considerably among expectant mothers, with certain predisposing factors contributing to the development of clinically significant symptoms. Women with a personal or family psychiatric history, including previous anxiety disorders, depression or post-traumatic stress disorder, demonstrate heightened vulnerability to PrA (2, 17, 18). Adverse obstetric history, including previous pregnancy loss and traumatic childbirth experiences, represents another significant risk domain and may create anticipatory anxiety regarding future perinatal outcomes (12, 16, 19). Current pregnancy complications, such as threatened preterm labour, gestational diabetes or abnormal screening results, constitute a particularly significant risk factor, as objective threats to maternal or foetal health may trigger or exacerbate anxiety symptoms (5, 10).

The quality and availability of social support emerge as a critical protective or risk factor, with insufficient partner and family support contributing to elevated anxiety levels, while robust support systems appear to buffer against its development (11, 20). Socioeconomic disadvantage, encompassing financial insecurity, unemployment and limited educational attainment, correlates with increased PrA through multiple pathways, including reduced access to healthcare and heightened life stressors. A history of traumatic experiences, including physical or sexual violence and childhood abuse, significantly elevates the risk of PrA, as pregnancy and childbirth may trigger reactivation of trauma-related fears concerning bodily autonomy and physical vulnerability (5).

Recognition of these risk factors enables healthcare providers to implement risk-stratified screening and develop individualised care plans, facilitating timely intervention and potentially mitigating adverse outcomes associated with untreated PrA.

Untreated or unrecognised PrA carries serious health consequences for both mother and child. Research indicates that women experiencing high levels of anxiety during pregnancy are at increased risk of adverse perinatal outcomes, including preterm birth, lower birth weight, and higher rates of obstetric interventions (7–10, 21) It should be noted that many studies reporting associations between prenatal anxiety and adverse perinatal outcomes have employed general anxiety measures rather than pregnancy-specific instruments such as the PRAQ-R2. Whether these associations are equally robust when anxiety is assessed as a pregnancy-specific construct warrants cautious interpretation. Furthermore, PrA is associated with reduced likelihood of initiating and maintaining breastfeeding (22), as well as an elevated risk of developing postpartum depression (PPD) and postpartum post-traumatic stress disorder (PPTSD) (23, 24). These findings underscore the importance of early identification and implementation of effective interventions to minimise the risk of serious consequences for maternal and child health.

#### 1.1.3 Diagnosis and measurement of pregnancy-related anxiety

The diagnosis of PrA represents a significant clinical challenge, as a moderate level of concern during pregnancy remains a physiological phenomenon, and formal diagnostic criteria are absent from DSM/ICD classifications. Emerging proposals in the literature have begun to advocate for the inclusion of PrA categories within future diagnostic frameworks, reflecting the growing body of evidence supporting the clinical distinctiveness of PrA (2, 4). Obstetric healthcare providers focus on the intensity, persistence, and functional consequences of anxiety symptoms.

A situation in which a pregnant woman experiences chronic, intense anxiety that interferes with activities of daily living or significantly diminishes quality of life may suggest the presence of PrA at a level requiring clinical intervention. An additional indicator of elevated PrA may be the patient’s expressed preference for operative delivery in the absence of objective medical indications. The diagnostic process for PrA is based on the identification of disproportionately elevated anxiety specific to pregnancy and childbirth, utilising both clinical criteria—assessing the intensity and functional consequences of anxiety—and validated screening instruments dedicated to the pregnant population.

The most frequently globally used measure is the Pregnancy-Related Anxiety Questionnaire—Revised 2 (PRAQ-R2) (25, 26), followed by the Pregnancy-Related Anxiety Scale (PRAS) (27), and the Cambridge Worry Scale (CWS) (28). Awareness of this issue is growing—numerous clinical guidelines currently recommend routine screening for anxiety disorders in pregnant women, supporting the integration of screening into standard antenatal care (6, 29, 30).

#### 1.1.4 Polish context

PrA is strongly influenced by sociocultural context, including social norms, beliefs about childbirth, healthcare accessibility, and stigma related to mental health. Cultural differences have been shown to influence symptom expression, coping strategies, and help-seeking behaviour, highlighting the need for culturally sensitive assessment tools and interventions (1).

Poland has one of the highest caesarean section rates in Europe. According to the European Perinatal Health Report, the caesarean section rate in Poland reached 44.4% between 2015 and 2019, compared to a European median of 26.0% (31). More recent national data from the Polish National Health Fund (NFZ) indicate a continuing upward trend, with caesarean section rates of 48.2% in 2023, 47.9% in 2024, and 48.4% in 2025 (32), placing Poland among the countries with the highest rates in Europe and the OECD. Single-centre data from a Polish tertiary referral centre further confirm this trend, reporting a caesarean section rate of 53.29% between 2020 and 2022, with mental health disorders identified as the most common non-obstetric indication, accounting for 50.68% of all non-obstetric caesarean sections (33). For context, the global average caesarean section rate was estimated at 21.1% in 2015, with Western Europe averaging 26.9% (34).

This alarming situation does not stem from medical necessity—research consistently demonstrates that a high level of fear of childbirth and the associated preference for operative birth are key contributing factors to these statistics (33, 35, 36).

Epidemiological analyses have demonstrated that the association between increasing caesarean section rates and reductions in maternal and neonatal mortality diminishes once these rates exceed approximately 19%. Given that caesarean section rates in Poland currently approach 48%, this suggests that non-medical factors, including PrA and fear of childbirth, may significantly influence delivery preferences, particularly among women at low obstetric risk (30, 34).

Research in Poland confirms that a preference for operative birth is strongly associated with elevated levels of fear of childbirth. Furthermore, a lack of alignment between the patient and the medical team regarding the birth plan has been identified as a significant risk factor for developing severe fear of childbirth. This psychological burden is reflected in clinical practice and underscores the fundamental importance of effective communication and respect for patient autonomy within the national perinatal care system (33, 36). Although fear of childbirth represents an important and clinically recognised concern, it constitutes only one component of pregnancy-related anxiety. PrA encompasses a broader range of concerns related to pregnancy, foetal health, maternal wellbeing, and early parenting, making PrA a more comprehensive target for preventive psychoeducational interventions.

The characteristics of the Polish perinatal care system constitute an additional factor intensifying the described problem. This system is marked by a high level of childbirth medicalisation, an institutional absence of midwifery care continuity, restricted availability of autonomous midwife-led care, and a deficit of practices ensuring full respect for patient autonomy and dignity. Qualitative research indicates that Polish women commonly encounter inadequate communication with healthcare providers, violations of privacy, excessive medical procedures and an inability to exert control over the labour and delivery process (37).

Globally, anxiety and related disorders during pregnancy represent a significant public health concern, estimated to affect between 20 and 40% of pregnant women, depending on the population studied, the type of anxiety assessed, and the sociocultural context (5). Pregnancy-related anxiety (PrA), as a construct distinct from generalized anxiety disorder, encompasses specific fears including concern for fetal health and fear of childbirth, which have been reported in up to 50% of pregnant women in large-scale surveys (18). Despite growing international evidence, research on PrA in the Polish population remains limited to single-centre pilot studies and psychometric validation work (26, 38), and no nationwide epidemiological study assessing the prevalence of PrA has been conducted to date. This represents a critical gap in current knowledge and underscores the importance of locally grounded research and intervention development.

A single-centre pilot study conducted among low-risk pregnant women in Poland found that more than half of participants (56%) scored at or above 30 points on the PRAQ-R2, indicating elevated PrA (38). This figure, while derived from a non-representative sample, provides the only available Polish-specific estimate of PrA prevalence and supports the clinical relevance of the present intervention within this population.

Additionally, women with greater financial resources are increasingly paying for private maternity care to ensure its continuity, establish a relationship with a midwife and minimise the risk of mistreatment—highlighting systemic shortcomings in publicly funded care. There is also evidence that insufficient knowledge and low confidence in pregnancy and childbirth-related knowledge among young Polish women are associated with higher levels of fear regarding childbirth and a preference for caesarean section, suggesting the need for prenatal education as a component of fear-reduction strategies (39).

Therefore, reducing the level of fear of childbirth in Poland is a matter of urgent public health concern. The high caesarean section rate carries significant health implications for both women and children, including increased short and long-term complication-related risks, prolonged recovery time, a higher risk of complications in subsequent pregnancies, and the so-called domino effect, where one caesarean delivery elevates the probability of the procedure being repeated in future births (30).

In its 2018 guidelines, WHO explicitly recommends the identification of pregnant women with high levels of childbirth-related anxiety and the implementation of interventions aimed at reducing this anxiety. The goal of these measures is to improve the quality of the birthing process among the population of low obstetric-risk pregnant women (6, 29, 30).

Several psychoeducational interventions targeting PrA and fear of childbirth have been evaluated internationally. These include structured group programmes combining life skills training and emotion regulation (13, 40), mindfulness-based approaches such as the Mindfulness-Based Childbirth and Parenting (MBCP) programme (15), brief individually-tailored interventions such as ACORN (41), and remote formats including tele-education delivered by midwives (42). A recent meta-analysis confirmed that antenatal education and midwifery-led care significantly reduce maternal anxiety and fear of childbirth across diverse settings (14). However, existing interventions have predominantly adopted group-based or remote formats, and most were conducted outside the Central and Eastern European context. Individually delivered, midwife-led psychoeducational programmes specifically targeting PrA in healthcare systems characterised by high medicalisation and fragmented maternity care such as Poland—remain largely untested.

The present protocol introduces an individualised, midwife-led psychoeducational intervention, tailored to the specific psychological needs and obstetric context of each participant. This approach is particularly relevant in healthcare systems characterised by fragmented maternity care and limited continuity of midwifery support, such as Poland. Additionally, the protocol integrates a structured multi-session format with a broad range of psychological and clinical secondary outcomes, enabling comprehensive evaluation of intervention effectiveness.

The present intervention is grounded in Hildegard Peplau’s Theory of Interpersonal Relations, which positions the therapeutic nurse–patient relationship as the core mechanism of care and anxiety reduction. Peplau’s model is particularly well-suited to pregnancy-related anxiety, as it emphasises the role of perceived control, emotional support, and structured interpersonal contact in promoting psychological wellbeing—factors consistently identified as predictors of anxiety in pregnant women. The theory has been applied across diverse clinical populations, including pregnant women, and provides a structured yet flexible framework for midwife-led psychoeducational interventions. A detailed description of how Peplau’s phases map onto the intervention sessions is provided in the Intervention section.

#### 1.1.5 Potential benefits and harms of the intervention

No serious harms are anticipated from participation in this psychoeducational intervention, as it is non-invasive and does not involve pharmacological treatment or clinical procedures. However, the intervention may involve discussion of anxiety-provoking topics, which could temporarily increase emotional distress in some participants. A predefined safety protocol with clear referral pathways to specialist psychological or psychiatric care will be implemented if needed. Adverse events will be monitored and reported to the Bioethics Committee of the Medical University of Gdańsk in accordance with standard procedures.

#### 1.1.6 Rationale for choice of comparator

Standard antenatal care was selected as the comparator because it represents current routine practice available to all pregnant women in Poland and constitutes the most clinically and ethically appropriate reference condition. A no-treatment control was considered ethically inappropriate given the elevated anxiety levels of enrolled participants. The addition of an informational booklet to standard care serves as a minimal active control, ensuring that all participants receive some structured information about childbirth preparation while maintaining a meaningful contrast with the full psychoeducational intervention. This approach allows for an assessment of the added benefit of the structured midwife-led programme over and above the information that could reasonably be provided within routine care.

### 1.2 Objectives

The primary objective of this study is to evaluate whether a tailored, individualised psychoeducational programme delivered by a trained midwife during the second and third trimester reduces the levels of PrA in low obstetric-risk Polish women, as measured by the PRAQ-R2 at 34–37 weeks of gestation.

The specific objectives are:

1. To evaluate the effect of the intervention on the levels of pregnancy-related anxiety at 34–37 weeks of gestation and at six or more weeks postpartum.
2. To determine whether a reduction in PrA is associated with improved birth outcomes, including mode of birth and rates of elective caesarean section without medical indication.
3. To assess the effect of the intervention on childbirth satisfaction and postpartum psychological wellbeing.
4. To monitor and report any unintended adverse effects of the intervention, including temporary increases in emotional distress during or following sessions.

### 1.3 Hypotheses

The following hypotheses will be tested:

*H1*: Women in the intervention group will demonstrate significantly lower levels of PrA at 34–37 weeks of gestation compared to women in the control group receiving standard antenatal care.

*H2*: Women in the intervention group will report significantly lower levels of depressive symptoms, higher childbirth satisfaction, and better maternal and neonatal health outcomes compared to women in the control group.

*H3*: Among all randomised participants, elevated PrA at baseline will be associated with a higher likelihood of caesarean section, less favourable attitudes towards breastfeeding, and poorer maternal and neonatal health outcomes, regardless of group allocation. H3 is an exploratory hypothesis examining the association between baseline anxiety and obstetric outcomes across both groups, independent of treatment allocation.

## 2 Methods

### 2.1 Patient and public involvement

No patients or members of the public were involved in the design, conduct, or reporting of this trial. This decision was made due to the exploratory nature of the study, limited resources, and the absence of an established patient advisory infrastructure at the study site. Future studies building on these findings will aim to incorporate patient and public involvement in intervention development and outcome selection.

### 2.2 Trial design

The study design is a two-arm, parallel-group, superiority randomised controlled trial (RCT) with a 1:1 allocation ratio. The study will evaluate the effectiveness of a structured, midwife-led psychoeducational intervention compared to standard antenatal care in reducing PrA among low-risk pregnant women in Poland. This protocol was developed in accordance with the SPIRIT 2025 Statement for protocols of clinical trials (43). Data will be analysed using intention-to-treat principles, insofar as outcome data are available.

### 2.3 Trial setting

This nationwide, multi-centre study will be conducted across Poland. Participants will be recruited from public antenatal clinics and outpatient departments, childbirth education schools, and through online platforms such as social media and parenting forums. Private maternity centres are excluded from recruitment to minimise selection bias. This multi-channel strategy ensures broad geographic reach and sample diversity. Intervention sessions will be delivered either in person at a location convenient for the participant or remotely via secure video conferencing. The study will be coordinated by the research team at the Medical University of Gdańsk.

### 2.4 Eligibility criteria

#### 2.4.1 Inclusion criteria

Women will be eligible for inclusion if they meet all of the following criteria: are at least 18 years of age, have a singleton pregnancy, are between 24 and 30 weeks of gestation at the time of recruitment, are fluent in Polish, are not receiving care exclusively at a private maternity centre, and score ≥30 points on the PRAQ-R2. Following a review of PRAQ-R2 score distributions across international validation samples—in which mean total scores ranged from approximately 22 points (SD 6.7) in a German third-trimester sample (44) to a median of 27 points (IQR 22–32) in a Chinese sample (45) and in light of Polish normative data reporting a mean score of approximately 31 points (38), this threshold was adopted for this study. It was established in a Polish single-centre pilot study and was associated with longer labour stages and higher rates of instrumental or emergency caesarean delivery (38). It is acknowledged that this cut-off captures women with elevated but potentially sub-clinical anxiety; however, given the absence of a universally validated diagnostic threshold for the PRAQ-R2 and given that most international adaptations recommend using the scale as a continuous measure (25, 45), this pragmatic threshold reflects the best available evidence in the Polish perinatal context.

#### 2.4.2 Exclusion criteria

Women will be excluded from the study if they meet any of the following criteria: absolute or strong medical indication for caesarean delivery (e.g., placenta praevia); multiple pregnancy; diagnosis of a severe psychiatric disorder (e.g., active psychosis, schizophrenia, bipolar disorder) that could interfere with informed consent or study participation; current pharmacological treatment for anxiety or depression, regardless of symptom severity, as concomitant psychotropic medication may confound the assessment of intervention effects; concurrent participation in another structured psychological intervention; or inability to commit to the study schedule.

#### 2.4.3 Eligibility criteria for intervention deliverers

Midwives delivering the intervention must meet all of the following criteria: hold a Master’s degree in midwifery, maintain current professional registration, and have a minimum of 2 years of clinical experience in obstetric or antenatal care. A minimum of 2 years was considered sufficient given that the intervention is protocol-based, does not require advanced psychotherapeutic qualifications, and is supported by a structured training programme completed by all participating midwives prior to delivery, covering the theoretical framework, session structure, psychoeducational techniques, and fidelity requirements.

Four to five midwives will be trained prior to trial commencement. Training objectives include: understanding Peplau’s theoretical framework; delivering the six-session protocol; applying anxiety management techniques; completing session logs; and recognising referral criteria. Competence will be confirmed via an observed practice session assessed by the principal investigator before each midwife proceeds to trial delivery. As fidelity is assessed by a single supervisor, formal inter-rater reliability testing is not applicable; this is acknowledged as a limitation.

### 2.5 Intervention and comparator

#### 2.5.1 Control group

Women in the control group will receive standard antenatal care as is typical in Poland, comprising: antenatal education in accordance with the Polish Organizational Standard of Perinatal Care (46), covering pregnancy, childbirth, puerperium and social support; routine obstetric appointments and ultrasound examinations; and hospital care during and after childbirth provided by designated hospital staff. In addition, women in the control group will receive an informational booklet on preparing for childbirth, developed specifically for this trial by midwives from the research team. The booklet covers key topics including the physiology of labour and birth, pain management options, hospital admission procedures, postpartum care, and newborn care. It is designed to provide factual information without therapeutic or psychoeducational components, thereby serving as a minimal active control condition that addresses ethical concerns while maintaining a meaningful contrast with the intervention.

Although standard antenatal education in Poland covers some overlapping topics (e.g., childbirth physiology), it is delivered in a group format without individualised psychological assessment or anxiety-focused components. The intervention differs fundamentally in being individually tailored to each participant’s anxiety profile and grounded in a structured therapeutic framework. To monitor the integrity of the control condition, participants will be asked at each follow-up assessment to report the content and format of antenatal education received; this information will be recorded and included as a covariate in sensitivity analyses if meaningful variability is identified.

Potential contamination between groups is addressed in detail in the Concomitant Care section, including measures to minimise cross-group communication and a planned sensitivity analysis.

#### 2.5.2 Intervention

Women in the intervention group will receive the same standard antenatal care and informational booklet as the control group, in addition to an individual psychoeducational programme led by a trained midwife. The intervention is advisory and educational in nature, with the aim of providing psychological support, reducing anxiety, and increasing the expectant mother’s sense of control and competence. It is based on communication skills, empathy, obstetric knowledge, and the application of simple psychoeducational and relaxation techniques. The intervention will be conducted between the 24th and 34th weeks of pregnancy in the form of individual sessions, which can take place in person or via secure video conferencing, depending on participant preference. The programme typically comprises four to six sessions of approximately 60 min each, held every one to 2 weeks. Women recruited toward the end of the eligible window (i.e., at 29–30 weeks of gestation) can complete the minimum required four sessions prior to the 34-week primary outcome assessment by attending one to two sessions per week, which is consistent with the standard frequency and does not represent an undue burden.

As described in the Introduction, the intervention is grounded in Peplau’s Theory of Interpersonal Relations (47, 48). This framework is particularly well-suited to PrA, which is strongly predicted by low perceived control and insufficient support from healthcare providers (49). Peplau’s four sequential phases map directly onto the intervention sessions: Orientation (Session 1), Identification (Sessions 2–3), Exploitation (Sessions 4–5), and Resolution (Session 6), with the midwife acting as teacher, counsellor, and surrogate throughout, involving the partner or family where appropriate (50, 51).

The intervention consists of six structured sessions, each with defined objectives and key activities, adaptable to the individual needs of the participant:

*Session 1*: Initial assessment and relationship building aims to establish contact, identify sources of anxiety and measure the baseline level of anxiety. In the introductory part, the midwife presents the purpose and principles of the intervention, ensuring confidentiality and an atmosphere of trust. Next, she conducts a diagnostic interview with open-ended questions regarding the patient’s fears, experiences and expectations, for example: “What are you most afraid of regarding childbirth?,” “Do you have previous childbirth experiences?,” “How do you remember them?,” “How does anxiety affect your daily functioning?.” At the end of the session, the midwife discusses and agrees upon the topics of subsequent sessions with the participant and provides educational materials, such as a programme leaflet and a notebook for recording thoughts and questions.

*Session 2*: Education on the course of childbirth is focused on increasing knowledge about the physiology of childbirth and verifying myths and misconceptions. The midwife discusses the stages—first, second and third—of childbirth in detail, explaining the symptoms, duration of each phase and standard medical procedures used at each stage (examinations, CTG monitoring, etc.). During the conversation, she addresses specific fears revealed earlier by the patient, providing comprehensive answers and correcting inaccurate information. In the case of multiparous women, a detailed interview is conducted regarding previous births, identifying difficult experiences and jointly seeking solutions and strategies with the midwife. The session is supported by materials such as brochures on the course of childbirth, anatomical diagrams and recommended educational videos.

*Session 3*: Methods of pain relief and stress management techniques is aimed at familiarising the patient with pharmacological and non-pharmacological methods of pain relief and teaching relaxation techniques. The midwife begins by explaining the physiology of labour pain and its role in the birthing process. She then presents non-pharmacological methods, such as breathing exercises, relaxation techniques, massages, labour positions, hydrotherapy or transcutaneous electrical nerve stimulation. Pharmacological methods are also discussed, including epidural anaesthesia, nitrous oxide and opioid medications, explaining their indications, benefits and potential limitations in detail. In the practical part, breathing and relaxation techniques are practised together with the patient; a companion may participate in the session at the patient’s request. At the end, the patient receives materials such as breathing exercise instructions, a list of pain relief methods and relaxation recordings. Partner participation will be documented in the session log and included as a covariate in sensitivity analyses if the frequency of attendance proves meaningful.

*Session 4*: Birth plan and communication with medical staff is focused on developing an individual birth plan and enhancing assertive communication skills with medical staff. The midwife introduces the concept of a birth plan as a document expressing the patient’s preferences and expectations regarding childbirth—for example, regarding labour positions, pain relief methods, the presence of a companion and skin-to-skin contact after birth. Together with the patient, she completes a birth plan form, discussing each option and providing objective information. The second part of the session is communication training—the midwife teaches the patient how to express her needs, ask questions and communicate assertively with the healthcare team. Specific phrases and conversation scenarios are practised, and the patient receives supporting materials, such as the completed birth plan, a leaflet on patient rights and a list of helpful questions for the staff.

*Session 5*: Anxiety management techniques and mental preparation for childbirth is aimed at equipping the patient with an individual set of psychological strategies for coping with anxiety before and during childbirth. The midwife begins by assessing the patient’s current emotional state and any progress made. She then deepens the discussion of relaxation and breathing techniques, introduces elements of mindfulness training, and works on the patient’s negative thoughts and beliefs—jointly developing positive affirmations and alternative, realistic thoughts. The next element is guiding a visualisation of a positive birth experience, aimed at desensitising anxiety and creating a constructive scenario in the patient’s imagination.

*Session 6*: Birth plan finalisation and Q&A concludes the intervention. The midwife summarises the entire process, reinforces the patient’s sense of competence and, if necessary, discusses options for further support. The patient receives a set of reinforcement materials. The PRAQ-R2 is administered at the last completed session to assess programme effectiveness, which may be Session 4, 5, or 6 depending on the number of sessions completed by the participant.

The intervention programme is flexible and can be adapted to the individual needs of the patient—both in terms of the number of sessions, topics, and format (with partner participation or independently). The intervention constitutes comprehensive educational support aimed at transitioning from anxiety to a sense of preparedness, control and inner peace ahead of the upcoming childbirth. An overview of the session structure, objectives and key activities is presented in Table 1.

**Table 1:** Intervention flow: objectives and activities per session.

Session	Objective	Key activities
1	Anxiety assessment and building rapport	Diagnostic interview, anxiety measurement (PRAQ-R2), session planning
2	Childbirth education	Discussion of labour stages, medical procedures, myth correction
3	Pain relief and stress management	Breathing and relaxation techniques, review of pain relief options
4	Birth plan and communication	Creating birth plan, assertive communication training with staff; final anxiety measurement (PRAQ-R2), if last session
5	Mental preparation and closure	Mindfulness, affirmations, positive visualisation; final anxiety measurement (PRAQ-R2), if last session
6	Birth plan finalisation and Q&A	Review of coping techniques, birth plan finalisation, Q&A, contact plan; final anxiety measurement (PRAQ-R2), if last session

#### 2.5.3 Intervention fidelity

Fidelity monitoring will be conducted by the principal investigator throughout the trial. Given the sensitive and confidential nature of the psychoeducational sessions, audio or video recording will not be used; instead, fidelity will be assessed through two complementary methods. First, each delivering midwife will complete a structured session log immediately after every session, documenting delivery of required content elements, techniques used, and any deviations from the protocol. Second, the principal investigator will conduct individual supervision meetings with each delivering midwife, during which session logs will be reviewed against a structured fidelity checklist operationalising the objectives and key activities defined in Table 1. Acceptable adherence is defined as completion of ≥80% of required content elements per session. If adherence falls below this threshold, the midwife will receive structured feedback and, where necessary, booster training before continuing delivery. All protocol deviations will be recorded and reported in the final manuscript.

#### 2.5.4 Criteria for discontinuing or modifying the intervention

The intervention may be discontinued or modified for an individual participant in the following circumstances: withdrawal of informed consent at any point during the trial; emergence of a severe psychiatric condition requiring specialist treatment (e.g., acute psychosis, severe depressive episode); clinically significant deterioration in psychological wellbeing as assessed by the delivering midwife and confirmed by the principal investigator; initiation of pharmacological treatment for anxiety or depression during the trial period; obstetric complications requiring hospitalisation or precluding participation; preterm birth prior to completion of the intervention; or inability to continue participation due to unforeseen personal circumstances.

In cases of discontinuation, the participant will be referred to appropriate clinical or psychological services where indicated. All outcome data collected prior to discontinuation will be retained and included in the intention-to-treat analysis. Participants who discontinue the intervention but remain willing to complete follow-up assessments will be encouraged to do so.

#### 2.5.5 Strategies to improve adherence and monitoring of adherence

Adherence will be supported through flexible scheduling in person or via secure video conferencing, provision of written session summaries and educational materials after each meeting, reminder messages prior to scheduled sessions, and telephone or message contact between sessions to maintain engagement. Partner or family involvement is encouraged where appropriate. Adherence will be monitored via a standardised session log maintained by the delivering midwife, recording the number, duration, and format of completed sessions. For the per-protocol analysis, adequate adherence is defined as completion of at least four of the six planned sessions. Reasons for non-attendance or early discontinuation will be recorded and reported.

#### 2.5.6 Concomitant care

Participants in both groups will continue to receive all routine antenatal care throughout the trial period. No concomitant structured psychological interventions or pharmacological treatments for anxiety or depression are permitted, as these constitute exclusion criteria. Use of other antenatal education resources, such as childbirth preparation classes or online informational materials, is not restricted but will be recorded at each follow-up assessment to allow for adjustment in sensitivity analyses.

The authors acknowledge that the multi-centre, multi-channel recruitment strategy—particularly the use of social media platforms and parenting forums—introduces a risk of contamination. Control participants may come into contact with intervention participants through the same online channels and consequently seek out psychoeducational support independently. This could result in an underestimation of the intervention effect. To minimise this risk, all participants will be asked at enrolment not to discuss study details with other enrolled women. At each data collection time point, control group participants will be asked whether they have used any form of psychological or psychoeducational support beyond standard antenatal care; this information will be recorded and included as a potential confounding variable in a sensitivity analysis.

### 2.6 Outcomes

#### 2.6.1 Primary outcome

The primary outcome is the levels of PrA, measured using the total score and three subscale scores of the Polish adaptation of the PRAQ-R2 (26). The total score ranges from 10 to 50 points. The three subscales are: Fear of Giving Birth (items 1, 2, 6; range 3–15), Worries about Bearing a Handicapped Child (items 4, 9, 10, 11; range 4–20), and Concerns about Appearance (items 3, 5, 7; range 3–15). The analysis metric is the change in total and subscale scores from baseline (Time I) to post-intervention assessment (Time II, 34–37 weeks of gestation), expressed as continuous variables. The method of aggregation is the mean score compared between groups.

#### 2.6.2 Secondary outcomes

Anxiety and depressive symptoms will be assessed using the PHQ-4 total score (range 0–12) and two subscale scores: PHQ-2 for depressive symptoms (items 1–2; cut-off ≥3) and GAD-2 for anxiety symptoms (items 3–4; cut-off ≥3). The metric is the change from baseline (Time I) to postpartum assessment (Time III, ≥6 weeks postpartum), with mean scores compared between groups.

Childbirth-related PTSD symptoms will be assessed prenatally using the PTSD-8 total score (range 0–24; no universally established cut-off in the original instrument) at baseline (Time I), and postnatally using the PPQ-II total score and subscale scores at Time III. Mean final values will be compared between groups.

Childbirth satisfaction will be measured using the BSS-R total score at Time III, with mean final values compared between groups.

Perceived social support will be assessed using the MSPSS total score (range 12–84) and three subscale scores (Significant Other, Family, Friends; range 4–28 each) at baseline (Time I) and postpartum (Time III), with mean scores compared between groups.

Infant feeding attitudes will be measured using the IIFAS total score at baseline (Time I) and reported descriptively.

Mode of birth and obstetric outcomes will be collected via the Custom Postpartum Questionnaire (Part B) at Time III and reported as frequencies and proportions.

Although all instruments will be administered in full, not all subscales or total scores will be analysed within the scope of this trial; some data will be reserved for secondary research questions and future analyses. Detailed descriptions of all measurement instruments, including psychometric properties of Polish adaptations, are provided in section 2.12 Data Collection Methods. Formal permissions for the use of all Polish adaptations have been obtained from the respective copyright holders prior to the commencement of the study.

### 2.7 Harms

Given the non-invasive nature of the intervention, no serious adverse events are anticipated. An adverse event is defined as any unintended negative psychological or physical experience reported by a participant during or following a session, including clinically significant increases in anxiety or acute emotional distress. Adverse events will be monitored by the delivering midwife at each session using the standardised session log and reported to the principal investigator within 24 h. Participants experiencing clinically significant deterioration will be referred to appropriate services in accordance with the safety protocol described in section Criteria for Discontinuing or Modifying the Intervention.

In cases where a participant develops severe anxiety, acute distress, or suicidal ideation, the delivering midwife will immediately notify the principal investigator, discontinue participation, and refer the participant to specialist psychiatric services. All such events will be reported to the Bioethics Committee of the Medical University of Gdańsk.

### 2.8 Participant timeline

**Figure 1:**
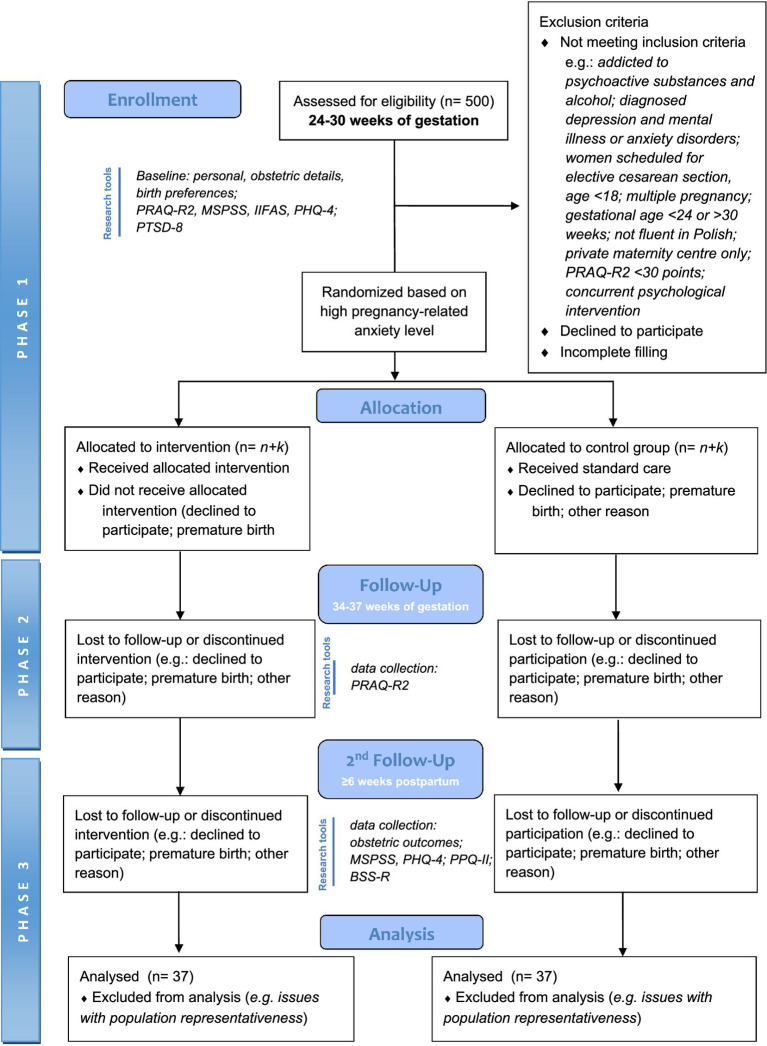
CONSORT flow diagram.

The schedule of enrolment, interventions, and assessments is presented in Figure 1 and Table 2. Eligible participants will be enrolled and randomised between 24 and 30 weeks of gestation (Time I). The intervention will be delivered between 24 and 34 weeks of gestation. The primary outcome assessment will be conducted at 34–37 weeks of gestation (Time II). The postpartum assessment will be conducted at six or more weeks after delivery (Time III). The duration of recruitment will continue until the target sample size of 37 participants per group is achieved.

**Table 2:** Schedule of enrolment, interventions and assessments (SPIRIT 2025).

Timepoint	Enrolment	Intervention period	Follow-up	Close-out
	Time I 24–30 weeks of gestation	24–34 weeks of gestation	Time II 34–37 weeks of gestation	Time III ≥6 weeks after delivery
Enrolment				
Eligibility screening	X			
Informed consent	X			
Randomisation	X			
Intervention				
Psychoeducational programme (Sessions 1–6)	X	X → → → X		
Standard antenatal care + booklet	X	X → → → X	X	X
Assessments				
Sociodemographic and clinical data (Part A)	X			
PRAQ-R2 (primary outcome)	X		X	
PHQ-4	X			X
PTSD-8	X			
MSPSS	X			X
IIFAS	X			
PPQ-II				X
BSS-R				X
Obstetric outcomes (Part B)				X

PP, postpartum; PRAQ-R2, Pregnancy-related anxiety questionnaire-revised 2; PHQ-4, Patient health questionnaire-4; PTSD-8, Post-traumatic stress disorder questionnaire; MSPSS, Multidimensional scale of perceived social support; IIFAS, Iowa infant feeding attitude scale; PPQ-II, Perinatal post-traumatic stress disorder questionnaire-II; BSS-R, Birth satisfaction scale-revised.

### 2.9 Sample characteristics and size

Sample size planning was informed by the Polish validation study of the PRAQ-R2, from which an anticipated 10% reduction in the mean score (3.27 points) and variability estimates for repeated measurements were derived. In this two-group randomised trial, the primary comparison will be the between-group difference in post-intervention PRAQ-R2 adjusted for baseline score, analysed using a two-sided significance level (*α*) of 0.05. Based on these assumptions, G*Power 3.1 indicated a minimum of 31 participants per group to achieve 80% statistical power. It should be noted that no MCID has been established for the PRAQ-R2 (26, 38). The 3.27-point planning assumption was derived from 10% of the Polish normative mean and additionally verified against distribution-based criteria: the anticipated difference approximates 0.5 SD of the total score [SD ≈ 8.56; (38)], a widely accepted proxy for minimal important difference in the absence of anchor-based estimates. This limitation is acknowledged; the present trial will contribute effect size data to inform future MCID derivation.

Because this is the first evaluation of an intensive, individually delivered midwife-led psychoeducational intervention in this setting, the final recruitment target of 74 women (37 per group) was retained as a pragmatic sample size balancing methodological considerations and intervention deliverability. The study is intended to provide an initial estimate of treatment effect and variance, and to inform the design of a future larger trial. An additional 20% was incorporated into the recruitment target to account for anticipated dropout, loss to follow-up, and incomplete outcome data (52, 53).

To achieve this, approximately 500 women will be screened in the second trimester to identify the eligible cohort with elevated PRAQ-R2 scores. This estimate reflects the expected prevalence of elevated PrA in the target population—a Polish pilot study found that approximately 56% of low-risk pregnant women scored ≥30 points on the PRAQ-R2 (26), combined with additional attrition anticipated from non-response, incomplete questionnaire completion, failure to meet remaining eligibility criteria, and non-consent prior to randomisation. Sample size planning was performed using G*Power 3.1.

### 2.10 Recruitment

Recruitment will be conducted by research midwives across three settings: antenatal clinics and outpatient departments, Lamaze classes, and via targeted advertisements on social media platforms and parenting forums. A convenience sampling approach across multiple recruitment settings will be used, followed by eligibility screening and random allocation among eligible participants.

Women expressing interest will receive a link to the study information sheet and the LimeSurvey platform. After reviewing the information and providing electronic informed consent, participants will complete the baseline assessment including the PRAQ-R2 and sociodemographic questionnaire. The LimeSurvey system has been configured so that the final PRAQ-R2 score is visible exclusively to the research team and not to participants, preventing unnecessary distress and allowing for objective eligibility assessment. Women whose PRAQ-R2 score meets or exceeds the predefined threshold will be contacted by telephone within 1 week to confirm willingness to participate and verify inclusion criteria. Women who provide consent at this stage will be formally enrolled in the trial.

To monitor recruitment balance across settings, the source of recruitment will be recorded for each participant at enrolment. Descriptive analyses will examine potential differences in baseline characteristics between women recruited through different channels, and recruitment source will be included as a covariate in sensitivity analyses if meaningful imbalances are identified.

### 2.11 Randomisation and blinding

Allocation to study groups at a 1:1 ratio will be performed using block randomisation with variable block sizes, computer-generated by a researcher not involved in recruitment. Allocation concealment will be ensured through sequentially numbered, opaque, sealed envelopes. The recruiting midwife will open the next consecutive envelope only after obtaining the participant’s final informed consent. Blinding of participants and intervention-delivering midwives is not feasible given the nature of the psychoeducational programme, consistent with standard practice in behavioural RCTs. Since all outcome questionnaires are self-completed via a secure online platform, no assessor is present at any data collection time point, which substantially mitigates assessment bias. The researcher responsible for data management and the data analyst will both be blinded to group allocation throughout the analysis phase, using coded group labels. Unblinding will only occur if authorised by the principal investigator for safety or ethical reasons and will be formally documented.

### 2.12 Data collection methods

All outcome measures will be collected via self-report questionnaires completed electronically by participants through the LimeSurvey platform at three time points: baseline (Time I, 24–30 weeks of gestation), post-intervention (Time II, 34–37 weeks of gestation), and postpartum (Time III, ≥6 weeks after delivery). No investigator-administered measurements will be performed. The data collection schedule is presented in Table 2.

To promote data quality, the LimeSurvey platform has been configured to prevent submission of incomplete questionnaires, to flag out-of-range responses, and to present items in a fixed order. All questionnaire links will be sent directly to participants via secure electronic communication. Data will be entered automatically into a password-protected database accessible only to the research team.

The following validated instruments will be used:

#### 2.12.1 Time I—prenatal assessment

The following instruments will be utilised for data collection during pregnancy:

1. Custom Prenatal Questionnaire (Part A): A self-report form designed to collect sociodemographic and clinical background information.
2. Iowa Infant Feeding Attitude Scale (IIFAS) (54), Polish adaptation (55): A 16-item self-report scale used to assess women’s attitudes and beliefs towards breastfeeding and formula feeding, to predict the choice of feeding method and intended duration of breastfeeding. The Polish adaptation demonstrated acceptable psychometric properties in a sample of 401 postpartum women, with satisfactory internal consistency (Cronbach’s *α* = 0.725), confirmed two-factor structure accounting for 31.18% of variance, and discriminative power coefficients above 0.2 for all items. Subscales were strongly correlated with the total score (*r* = 0.803 for both subscales) (55).
3. Multidimensional Scale of Perceived Social Support (MSPSS) (56), Polish adaptation (57): A 12-item self-report tool for measuring perceived social support from three sources: family, friends, and a significant other. The Polish adaptation demonstrated satisfactory psychometric properties in a sample of 1,322 adults, with confirmed three-factor structure (RMSEA = 0.065; *χ^2^*/df = 2.957) and high internal consistency (Cronbach’s *α* = 0.893 for the total score; *α* = 0.931 for Friends; *α* = 0.915 for Family; *α* = 0.865 for Significant Other), as well as satisfactory temporal stability and convergent validity (57).
4. Pregnancy-Related Anxiety Questionnaire—Revised 2 (PRAQ-R2), Polish adaptation (26). The primary outcome measure, the PRAQ-R2, is a 10-item questionnaire that covers three subscales of anxiety specific to pregnancy and childbirth: (1) fear of childbirth, (2) worries that the child will be physically or mentally disabled, and (3) concern about one’s own appearance during pregnancy. The overall and factor scores demonstrate good reliability and validity regardless of parity. The Polish adaptation of the PRAQ-R2 demonstrated good psychometric properties in a sample of 175 Polish pregnant women. Confirmatory factor analysis confirmed the original three-factor structure (CFI = 1.00; TLI = 1.00; RMSEA < 0.001), and internal consistency was satisfactory across all subscales and the total score (Cronbach’s *α* ranging from 0.68 to 0.94). Convergent validity was confirmed through significant correlations with the Hospital Anxiety and Depression Scale (HADS) and the Visual Analogue Scale (VAS) (26).
5. Patient Health Questionnaire-4 (PHQ-4), Polish version: A short questionnaire used in screening in primary healthcare practice and clinical settings for examining anxiety and depressive symptoms. The Polish version demonstrated good psychometric properties in a large community sample of Polish adults (*n* = 1,115), with acceptable internal consistency (Cronbach’s *α* = 0.74 for the anxiety subscale and *α* = 0.77 for the depression subscale), confirmed two-factor structure, and measurement invariance across age and gender groups (58).
6. Post-Traumatic Stress Disorder Questionnaire (PTSD-8), Polish (59): The PTSD-8 questionnaire contains questions about stress reactions manifested as intrusion (four items), avoidance (two items) or hyperarousal (two items). It is both a diagnostic tool for PTSD and an assessment tool to evaluate the severity of this syndrome, its determinants and health consequences. The Polish adaptation demonstrated high reliability (Cronbach’s α = 0.891, McDonald’s *Ω* = 0.890) and acceptable construct validity based on confirmatory factor analysis of an adjusted single-factor model (CMIN/DF = 4.944; NFI = 0.976; RFI = 0.955; IFI = 0.981; TLI = 0.964; RMSEA = 0.073, 90% CI: 0.057–0.090). Factor loadings ranged from 0.613 to 0.760, and average variance extracted (AVE) was 0.489 (59).

#### 2.12.2 Time II—post-intervention assessment

The second phase of data collection will consist solely of a follow-up measurement using the PRAQ-R2 to assess changes in the primary outcome.

#### 2.12.3 Time III—postpartum assessment

The following instruments will be utilised for data collection after delivery:

1. Custom Postpartum Questionnaire (Part B): a self-report form designed to collect supplementary interview data and clinical information following childbirth.
2. Perinatal Post-Traumatic Stress Disorder Questionnaire-II (PPQ-II), Polish translation (60, 61): a 14-item self-report questionnaire assessing childbirth-related PTSD symptoms across two domains: Avoidance/Intrusion and Arousal. The Polish adaptation demonstrated very good psychometric properties in a sample of 273 postpartum mothers, with high internal consistency (Cronbach’s *α* = 0.92 for the total score; *α* = 0.91 for Avoidance/Intrusion; *α* = 0.86 for Arousal), confirmed two-factor structure explaining 62% of total variance (KMO = 0.90), good convergent validity with the PTSD-8 (*r* = 0.43–0.85) and DASS-21 (*r* = 0.54–0.78), and excellent temporal stability over a 3-month interval (*r* = 0.90) (61)
3. MSPSS: as described above (Time I) (57)
4. Birth Satisfaction Scale-Revised (BSS-R), Polish (62, 63)
5. PHQ-4: As described above (Time I) (58)

To promote retention, reminder messages will be sent prior to each data collection time point, and research midwives will maintain contact with participants throughout the study period. Participants who discontinue the intervention but remain willing to complete follow-up assessments will be encouraged to do so. Where possible, outcome data from participants who withdraw will be retained for intention-to-treat analysis. Reasons for withdrawal and loss to follow-up will be recorded and reported.

### 2.13 Data management

All data will be collected electronically via LimeSurvey and stored in a secure database accessible only to the research team. Participants will be identified by anonymous study ID numbers; the linkage file will be stored separately and accessible only to the principal investigator. Data will be checked for completeness and out-of-range values prior to database lock. All study data will be retained for a minimum of 10 years on the institutional servers of the Medical University of Gdańsk, in accordance with GDPR. Anonymous data may be shared upon reasonable request, subject to approval by the principal investigator and the Bioethics Committee.

### 2.14 Statistical analysis plan

Analyses will be conducted according to randomised group allocation and will primarily follow the intention-to-treat principle, insofar as outcome data are available. The primary endpoint is PrA measured using the PRAQ-R2 at 34–37 weeks’ gestation. The primary analysis will compare post-intervention PRAQ-R2 scores between groups using analysis of covariance (ANCOVA), with treatment group as the main factor and baseline PRAQ-R2 score as a covariate. The primary outcome is assessed at two prenatal time points only: baseline and post-intervention. The postpartum assessment applies exclusively to secondary outcomes.

Secondary outcomes will be analysed using regression models appropriate to outcome type, with logistic regression for binary outcomes and linear regression for continuous outcomes. Secondary analyses will be considered supportive and exploratory; no formal adjustment for multiplicity is planned. Missing data will be described by study arm and time point. The primary analysis assumes data are missing at random (MAR); this assumption will be examined via sensitivity analysis, using a mixed-effects model for repeated measures applied to all available longitudinal data. Multiple imputation is not planned. The possibility of data missing not at random (MNAR) cannot be excluded and is acknowledged as a limitation. Stratification variables included in the randomisation will be entered as covariates in the primary ANCOVA model. A supportive per-protocol analysis will also be conducted among participants with observed primary endpoint data; in the intervention arm, this will additionally require completion of at least four intervention sessions. Two pre-specified sensitivity analyses will be conducted: (1) a linear mixed-effects model including midwife as a random effect, to account for potential clustering by intervention deliverer; and (2) a mixed-effects model for repeated measures applied to all available longitudinal data under a missing-at-random assumption, to assess the robustness of findings in the presence of incomplete data.

The mode of intervention delivery (in-person versus remote) will be recorded. As the trial is not powered for formal moderator analyses, any comparison by delivery mode will be considered exploratory and interpreted cautiously.

### 2.15 Data monitoring and trial monitoring

No independent data monitoring committee (DMC) has been established for this trial, given the small sample size, the non-invasive and low-risk nature of the psychoeducational intervention, and the absence of planned interim analyses. Safety monitoring and trial conduct will be overseen by the principal investigator throughout the study period, including regular review of recruitment progress, adherence to the intervention protocol, completeness of data collection, and adverse event reporting in accordance with the procedures described in section Harms.

## 3 Ethics

This trial was approved by the Bioethics Committee of the Medical University of Gdańsk (KB/367/2025, KB/20/2025, KB/20–80/2026) and will be conducted in accordance with the 1964 Declaration of Helsinki. Any important protocol amendments will be submitted to the Bioethics Committee prior to implementation and reported to the trial registry. Women meeting the eligibility criteria will receive written study information and will be encouraged to ask questions before deciding to participate. Electronic informed consent will be obtained from all participants prior to inclusion. Participants may withdraw at any time without justification or consequences for their clinical care. All personal data will be handled in accordance with GDPR. Adverse events will be monitored and reported as described in section Harms. No compensation or post-trial care provisions are applicable given the non-invasive nature of the intervention.

## 4 Discussion

This study protocol describes a planned randomised controlled trial aimed at evaluating the effectiveness of an individualised psychoeducational programme delivered by midwives. The programme is designed to reduce PrA and reduce the preference for caesarean section in the absence of medical indications among pregnant women in Poland who are at low risk of complications. The intervention is based on patient empowerment, education and emotional support, and aims to fill a significant gap in perinatal mental healthcare in a country where caesarean section rates are exceptionally high (64).

The rationale for this intervention is robust, supported by growing scientific evidence indicating that pregnancy-related anxiety constitutes a distinct and significant health concern. Unlike generalised anxiety, PrA focuses on specific aspects of pregnancy, such as foetal health, labour pain and maternal competence, making it particularly amenable to targeted psychoeducation. The intervention structure corresponds to recommended components of programmes designed to reduce fear of childbirth. It ranges from initial assessment and relationship-building, through education on childbirth physiology and pain management, to communication skills and birth planning. By equipping women with knowledge and practical coping strategies, the intervention aims to enhance their sense of control and self-efficacy, which are key psychological factors in mitigating PrA.

The selection of midwives to deliver the intervention is deliberate and holds particular significance in the Polish context. The Polish perinatal care system is characterised by a high degree of medicalisation and fragmentation, which often leaves women feeling disempowered regarding the course of their pregnancy and childbirth, thereby intensifying their anxiety. Midwives, owing to their specialised knowledge of physiological pregnancy and childbirth, are ideally positioned to provide continuity of care, woman-centred communication, and support that does not pathologize natural processes. Training midwives in structured psychoeducational techniques—without requiring advanced psychotherapeutic qualifications—enhances the intervention’s scalability and potential for integration into standard prenatal care pathways. This approach aligns with the guidelines proposed by the World Health Organization (WHO), which recommend non-pharmacological interventions to reduce unnecessary caesarean sections, particularly through addressing maternal anxiety (29).

This approach is further validated by recent evidence from Padilla et al., who demonstrated that online group prenatal education delivered by midwives significantly reduced fear of childbirth in women with high levels of fear (mean difference = 22.74 points, *p* < 0.001). Their intervention—based on self-care deficit theory and consisting of five weekly videoconferencing sessions—also showed trends towards improved obstetric outcomes, including higher rates of spontaneous labour onset (65). Importantly, this was among the first midwife-led trials to evaluate a comprehensive intervention spanning both prenatal and intrapartum periods, demonstrating the feasibility and effectiveness of midwife-delivered psychoeducational programmes in routine prenatal care settings. It should be noted that the effect size reported by Padilla et al. is not directly comparable to the effect estimate used in the present power calculation, as the two studies differ in measurement instruments, intervention format, and comparison conditions. The present trial was powered based on Polish normative PRAQ-R2 data, adopting a conservative estimate to ensure robustness of the sample size calculation.

The evaluation of this intervention may additionally contribute to reducing the rate of medically unjustified caesarean sections by addressing one of their key psychological determinants. Should the trial demonstrate effectiveness, the implications would be two-fold. First of all, it would provide an evidence-based, practical tool for addressing the rising caesarean section rates in Poland—a public health crisis. By reducing the anxiety-driven demand for elective surgical procedures, the intervention could contribute to lowering procedural rates to WHO-recommended levels, thereby reducing associated maternal and neonatal morbidity. Secondly, it would validate a care model that strengthens the role of midwives as autonomous providers of psychological support, potentially informing health policy changes towards more holistic and continuous midwifery-led care models in Poland.

The transferability of findings to other healthcare contexts should, however, be considered carefully. The present intervention is embedded within a specific Polish perinatal care context, characterised by high medicalisation, limited midwifery autonomy, and fragmented continuity of care. Countries with similar structural characteristics—including high caesarean section rates driven by non-medical factors, restricted access to midwife-led care, and comparable cultural attitudes towards childbirth—may represent the most appropriate settings for replication. By contrast, healthcare systems with well-established midwifery continuity models, routine psychosocial screening, or lower baseline anxiety levels may yield different effect sizes. Additionally, cultural norms surrounding mental health, help-seeking behaviour, and the perceived role of midwives in psychological support vary considerably across countries and should be accounted for when adapting this intervention to new contexts.

Should the trial fail to demonstrate a statistically significant reduction in PrA, this would itself constitute a scientifically valuable finding. A null result would suggest that the individualised midwife-led psychoeducational format, as implemented in this protocol, may not be sufficient to produce measurable anxiety reduction within the studied population and timeframe. Such a finding would prompt reconsideration of the intervention intensity, session frequency, or target population, and could redirect future research towards alternative formats, such as group-based interventions or those incorporating psychological specialist support. From a policy perspective, a null result would caution against large-scale implementation of this specific model without further evidence, while still supporting the broader agenda of integrating mental health screening into routine antenatal care in Poland.

The potential limitations of this study must be acknowledged. Reliance on self-report measures for the primary outcome, despite using a validated instrument (PRAQ-R2), introduces the possibility of reporting bias. The sample, recruited based on high PrA scores and Polish language proficiency, may not be fully representative of the entire pregnant population. Furthermore, intervention effectiveness may depend on the skills and consistency of individual midwives, despite standardised training and fidelity monitoring. Although variability in intervention delivery across midwives is acknowledged as a limitation, this will be statistically addressed by including midwife as a random effect in a mixed-effects model, thereby accounting for clustering within deliverers. Nevertheless, residual variability in therapeutic style, communication approach, and professional experience cannot be fully eliminated and may influence participant outcomes. The control group receiving “standard care” may experience varying levels of support, though provision of a basic informational booklet aims to partially address ethical concerns and somewhat standardise this aspect.

Several additional limitations should be acknowledged. First, the booklet-only control condition does not constitute an active comparator, which precludes conclusions about which specific components of the psychoeducational intervention are therapeutically active; a dismantling study would be required to address this question. Second, the primary outcome is assessed at 34–37 weeks of gestation, a period during which anxiety levels may naturally fluctuate due to proximity to childbirth, independent of any intervention effect; this third-trimester confound cannot be fully disentangled from intervention-related change. Third, the postpartum data collection window is defined as six or more weeks after birth, which may introduce heterogeneity in outcome measurement due to variability in the timing of assessments across participants; future studies should consider a more precisely defined postpartum assessment window. The primary outcome is assessed immediately following intervention completion, which does not permit evaluation of effect durability. An additional assessment at 38–40 weeks was considered but deemed unfeasible due to participant burden in late pregnancy and logistical constraints in scheduling prior to delivery; this is recommended as a priority for future trials.

Furthermore, the multi-channel recruitment strategy—encompassing public antenatal clinics, childbirth education schools, and online platforms—may result in a heterogeneous study population. Women recruited through different settings may differ systematically in health literacy, sociodemographic characteristics, and anxiety profiles. Although recruitment source will be recorded and included as a covariate in sensitivity analyses if meaningful imbalances are identified, residual differences between subgroups cannot be fully eliminated and may limit the generalisability of findings. The restriction to women scoring ≥30 on the PRAQ-R2—a pragmatic threshold based on Polish normative data rather than a validated clinical cut-off—limits generalisability to women with lower but potentially clinically relevant anxiety levels. Reasons for screening refusal will be recorded where possible and reported in the CONSORT flow diagram to allow assessment of selection bias.

Future research stemming from this trial could be focused on examining the long-term effects of the intervention on maternal mental health (e.g., postpartum depression, bonding), infant health outcomes and the sustainability of birth mode decisions in subsequent pregnancies. Cost-effectiveness analyses would be crucial for promoting wider implementation within the public healthcare system. Additionally, qualitative studies exploring the experiences of both women and midwives participating in the programme could provide valuable insights for refining the intervention and understanding its mechanisms of action.

## 5 Expected results

Based on evidence from comparable psychoeducational interventions and Polish normative PRAQ-R2 data, we anticipate that women in the intervention group will demonstrate a clinically meaningful reduction in PrA at 34–37 weeks of gestation compared to the control group. Secondary outcomes are expected to show improved childbirth satisfaction, lower rates of elective caesarean section without medical indication, and better postpartum psychological wellbeing. These expectations will be formally tested in the trial.

## 6 Conclusion

This RCT protocol presents a novel and contextually adapted approach to addressing the intertwined challenges of high PrA in Poland. By testing a midwife-delivered psychoeducational programme, the study addresses a clear clinical need while contributing to the broader scientific understanding of non-pharmacological interventions in perinatal mental health. If proven effective, this intervention has the potential to improve the psychological wellbeing of pregnant women, promote physiological childbirth and inform the evolution of more supportive and empowering perinatal care practices in Poland as well as in similar healthcare settings.

### Oversight

The study is overseen by the research team at the Division of Obstetric and Gynaecological Nursing, Medical University of Gdańsk. No independent steering committee or data monitoring committee has been established, given the small sample size and low-risk nature of the psychoeducational intervention. Trial monitoring will be conducted by the principal investigator in accordance with the procedures described in the Trial Monitoring section.

### Protocol and statistical analysis plan

The full trial protocol and statistical analysis plan are published as part of this manuscript. Any supplementary analytical details are available upon request from the corresponding author (a.krawczyk@gumed.edu.pl).

### Data sharing

The individual de-identified participant data, data dictionary, and statistical code generated during this study will not be publicly deposited in a repository. However, these materials are available upon reasonable request from the corresponding author, subject to compliance with applicable data protection regulations and approval by the Bioethics Committee of the Medical University of Gdańsk.

### Dissemination policy

The results of this trial will be submitted for publication in a peer-reviewed journal. Open access publication will be pursued where possible. Findings will also be presented at relevant national and international conferences. Upon completion of the study, participants who provided consent to be contacted will be informed of the key findings via the contact details collected at enrolment. No commercial dissemination is planned.
